# Pioglitazone Attenuates the Effects of Peripheral Inflammation in a Human In Vitro Blood–Brain Barrier Model

**DOI:** 10.3390/ijms232112781

**Published:** 2022-10-24

**Authors:** Gustavo Henrique Oliveira da Rocha, Rodrigo Azevedo Loiola, Marina de Paula-Silva, Fumitaka Shimizu, Takashi Kanda, Andrea Vieira, Fabien Gosselet, Sandra Helena Poliselli Farsky

**Affiliations:** 1 Faculty of Pharmaceutical Sciences, University of São Paulo, São Paulo 05508-900, Brazil; 2 Laboratoire de la Barrière Hémato-Encéphalique (LBHE), Faculté des Sciences Jean Perrin, Artois University, UR 2465, F-62300 Lens, France; 3 Department of Neurology and Clinical Neuroscience, Yamaguchi University, Ube 755-8505, Japan; 4 Faculty of Medical Sciences, Clinic of Gastroenterology, Department of Medicine, Irmandade da Santa Casa de Misericórdia de São Paulo, São Paulo 01221-020, Brazil

**Keywords:** blood–brain barrier, pioglitazone, peripheral inflammation, inflammatory bowel disease

## Abstract

Biological mediators secreted during peripheral chronic inflammation reach the bloodstream and may damage the blood–brain barrier (BBB), triggering central nervous system (CNS) disorders. Full-fledged human BBB models are efficient tools to investigate pharmacological pathways and mechanisms of injury at the BBB. We here employed a human in vitro BBB model to investigate the effects of either plasma from inflammatory bowel disease (IBD) patients or tumor necrosis factor α (TNFα), a cytokine commonly released in periphery during IBD, and the anti-inflammatory role of pioglitazone, a peroxisome proliferator-activated receptor γ agonist (PPARγ). The BBB model was treated with either 10% plasma from healthy and IBD donors or 5 ng/mL TNFα, following treatment with 10 µM pioglitazone. Patient plasma did not alter BBB parameters, but TNFα levels in plasma from all donors were associated with varying expression of claudin-5, claudin-3 and ICAM-1. TNFα treatment increased BBB permeability, claudin-5 disarrangement, VCAM-1 and ICAM-1 expression, MCP1 secretion and monocyte transmigration. These effects were attenuated by pioglitazone. Plasma from IBD patients, which evoked higher BBB permeability, also increased ICAM-1 expression, this effect being reversed by pioglitazone. Our findings evidence how pioglitazone controls periphery-elicited BBB inflammation and supports its repurposing for prevention/treating of such inflammatory conditions.

## 1. Introduction

The blood–brain barrier (BBB) is an essential component of central nervous system (CNS) physiology located at the blood microvessel level, being the interface regulating exchanges of molecules and cells between the brain and periphery. This barrier system is kept tightly impermeable to peripheral factors due to junctional proteins organized in tight (TJ) and adherens junctions (AJ) connecting endothelial cells. Under physiological conditions, the BBB protects the brain from toxins and inflammatory factors, finely regulates influx/efflux transport of molecules and prevents the passage of peripheral leukocytes into brain tissue [1,2].

Chronic peripheral inflammation arising from conditions such as lupus, asthma, rheumatoid arthritis, inflammatory bowel diseases (IBD) and even infections leads to increases in inflammatory mediators circulating in the bloodstream, such as cytokines and chemokines [3]. These inflammatory mediators when in contact with the BBB are known to disrupt endothelial cell homeostasis, modifying expression of junction proteins and facilitating transmigration of activated leukocytes into brain tissue [1]. In this context, the study of drugs aimed at attenuation of BBB inflammation triggered by peripheral inflammatory conditions, which in turn can prevent further neuroinflammation and can be used alongside other drugs for treatment of chronic peripheral diseases, has been largely neglected [4].

One such class of drugs comprises thiazolinediones, which are peroxisome proliferator-activated receptor γ (PPARγ) ligands. Long used for treatment of type II diabetes mellitus, these drugs have been studied in past years due to their anti-inflammatory actions, but thus far have not yet gone past clinical trials due to lack of understanding of their mechanisms and effects in their entirety, despite results of clinical studies having shown promise [5,6]. Studies have evidenced that PPARγ ligands can be useful not only in treating chronic peripheral inflammatory diseases, as they decrease disease activity and improve the effects of other anti-inflammatory drugs in arthritis models [7,8], but also in treating neurodegenerative disorders, as evidenced by their ability to stimulate neuron-induced degradation of ß-amyloid peptides in mouse models [9]. Among successfully commercialized thiazolinediones, pioglitazone and rosiglitazone have been employed for a whole decade from the year 2000 for treatment of type II diabetes, but rosiglitazone use has been discontinued in Europe and the United States of America in 2010 following large clinical trials evidencing increased cardiovascular risk due to its use. Pioglitazone has remained since then the only clinically relevant thiazolinedione and the most commonly PPARγ ligand studied for drug repurposing aimed at treating inflammatory disorders [10,11]. Still, thus far, no studies have reported any effects of PPARγ ligands, let alone pioglitazone, on the BBB addressing inflammation. Understanding of the anti-inflammatory mechanisms of pioglitazone on the BBB could incentivize its use as an adjuvant therapy aimed at reducing BBB damage and preventing onset of neuroinflammation during treatment of chronic peripheral inflammatory disorders.

IBD, a chronic debilitating condition comprising ulcerative colitis (UC) and Crohn’s disease (CD), is characterized by immune imbalance and continuous inflammation originated in the intestines and is one of such disorders where this approach for repurposing of PPARγ ligands would be feasible. Recent cohort studies have confirmed that IBD patients are much more likely to develop neurodegenerative diseases [12,13], and there are also experimental studies evidencing how peripheral inflammation during the progression of IBD links to neuroinflammation, as it has been shown in mice models of experimental colitis that there is increased migration of peripheral leukocytes into the brain, activation of microglia and loss of neurons, all such effects in association with BBB disruption [1,14]. All the while, PPARγ ligands have been shown to halt clinical progression of IBD at different stages in mice models [1,15,16,17] and have even reached clinical trials in human IBD patients [6,18], but associated mechanisms have yet to be further studied.

We here aimed in a fully human in vitro BBB model to investigate the actions of pioglitazone in restoring BBB homeostasis and preventing damage triggered by peripheral inflammatory factors, exploring such effects utilizing TNFα, a major cytokine released during peripheral inflammation, and plasma from IBD patients as a further source of peripheral inflammatory stimuli.

## 2. Results

### 2.1. The BBB Is Sensible to Peripheral Inflammatory Factors

Plasma was collected from 30 IBD patients of both sexes and of varied ages at different disease stages and from 30 healthy donors matched by age and sex (Table 1). Initial screening of inflammation-associated cytokines in patient plasma revealed no changes in cytokine levels other than a slight decrease in TNFα and MMP-9 levels in comparison to healthy donors, likely due to patients being treated with different anti-inflammatory drugs (Table 2).

In order to verify which BBB components would be affected by peripheral inflammatory factors found in donor plasma, BLECs were treated with such plasma; permeability coefficient of a small paracellular marker through BLECs and expression of junctional and inflammation-associated proteins were assessed.

**Figure 1:**
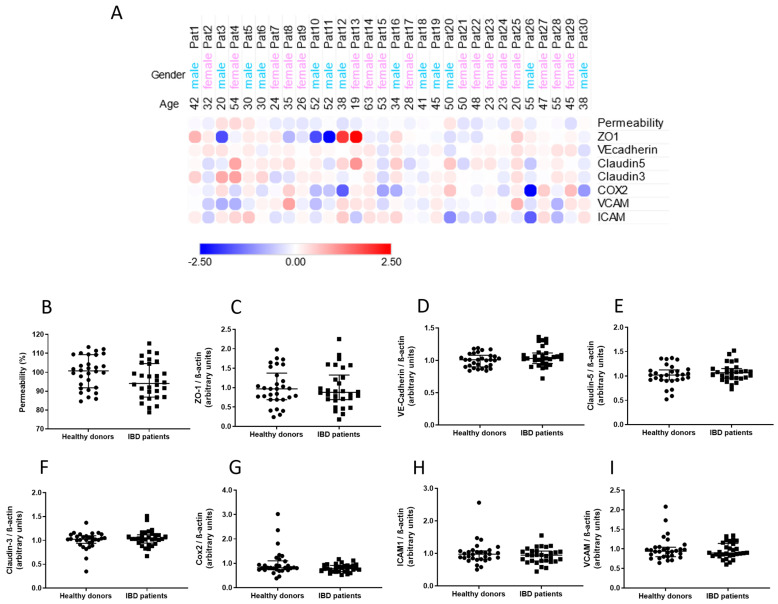
**Comparisons between healthy donors and patientsof parameters assessed in BLECs treated with donor plasma.** Plasma was obtained from n = 30 IBD patients and 30 healthy donors matched by age and sex. BLECs were treated with 10% plasma for 24 h and permeability to NaFlu, and expression of proteins by Western blotting were assessed. An initial comparative analysis of these parameters was carried out, and results are shown as a bubble heatmap evidencing log2 fold-changes of assessed parameters for IBD patients in comparison to healthy donors (log2 fold-change magnitude represented in the bar below the graph) (**A**). Further comparisons of paired data were carried out for all parameters assessed: permeability (**B**), expression of junction proteins (**C**–**F**) and expression of inflammation-related proteins (**G**–**I**); results are shown as medians alongside interquartile ranges. Comparisons were carried out via Wilcoxon signed rank test. Permeability value of controls = 0.55 ± 0.046 × 10^−3^ cm/min.

An initial and global observation of the assessed parameters revealed no obvious pattern of alterations induced by patient plasma on an individual-by-individual basis (Figure 1A). Further analysis between levels of the assessed parameters revealed that there were no statistically significant differences caused by treatment with patient plasma in comparison to treatment with plasma from healthy donors (Figure 1B–I).

**Figure 2:**
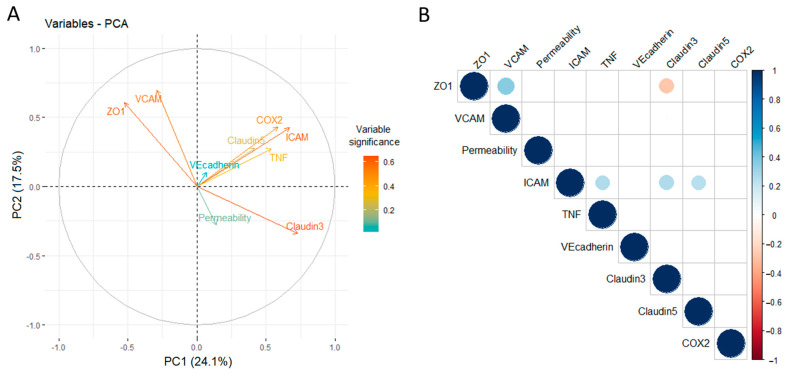
**Exploratory analysis of data generated from treatment of BLECs with donor plasma.** Plasma was obtained from n = 30 IBD patients and 30 healthy donors matched by age and sex. BLECs were treated with 10% plasma for 24 h and permeability to NaFlu, and expression of proteins by Western blotting were assessed. PCA of these parameters alongside TNFα levels in donor plasma was carried out, and loading plots of assessed parameters are shown (heat scale at the side of the graph indicate numerical significance to the model of the assessed parameters) (**A**). A correlation matrix correlating all assessed parameters was also generated (only correlations of *p* value < 0.05 are shown, this being represented by circles of larger size, the smaller the *p* value is; the heat-scale at the side of the graph indicates in blue whether significant correlations are positive or in red in case correlations are negative) (**B**).

However, further assessment of BLEC responses by PCA performed with data pertaining parameters from BLECs treated with plasma from either healthy donors or IBD patients revealed that TNFα levels in plasma, alongside expression of claudin-5, ICAM-1 and COX-2 by BLECs, contribute reasonably evenly and on a same directional vector to the variation of the PCA mathematical model, while expression of claudin-3, VCAM-1 and ZO-1 contribute in an unrelated manner to the model, and finally, permeability to NaFlu explains very little of the variation observed (Figure 2A). These findings indicate that the BBB model used is sensible to peripheral inflammatory factors, especially TNFα found in plasma, as response patterns as assessed by PCA can be identified even though differences induced by plasma among all donors is minimal, considering plotting of individual PCA values indicated no clustering (Supplementary Figure S1A). In order to confirm such observations, a correlation matrix with all assessed parameters was generated. TNFα levels in plasma of all donors correlate positively and significantly with expression of ICAM-1 and expression of claudin-5 and claudin-3 correlating in the same manner. Claudin-3 expression correlates negatively with ZO1 expression, but there is no correlation of any of these parameters with TNFα plasma levels or permeability values. These results confirm PCA observations and indicate that BLECs are sensible to TNFα levels in plasma of both healthy donors and IBD patients, which in turn likely modulate expression of ICAM-1, claudin-5 and claudin-3 while not influencing permeability, at least under the tested conditions (Figure 2B, Supplementary Figure S1B–G).

### 2.2. Pioglitazone Improves BBB Permeability under TNFα Treatment Independently of Alterations in Junctional Protein Expression

**Figure 3:**
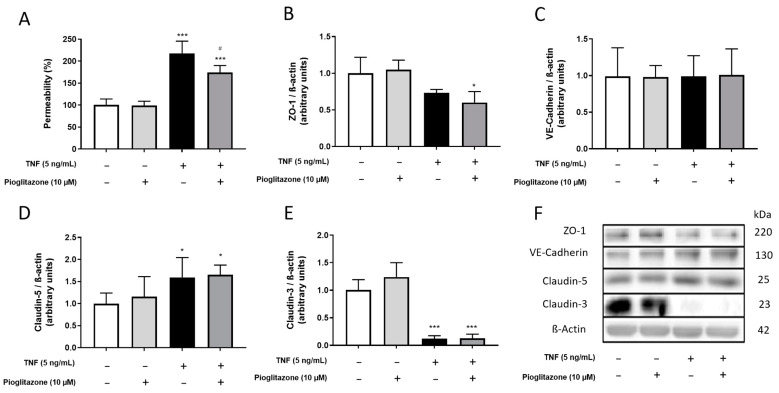
**Effects of pioglitazone on TNFα-induced permeability alterations and junctional protein expression.** BLECs were treated with 5 ng/mL of TNFα for 24 h following pre-treatment with 10 µM pioglitazone for 24 h. Permeability to NaFlu (**A**) and expression of junctional proteins ZO1, VE-cadherin, claudin-5 and claudin-3 by Western blotting (**B**–**F**) were assessed. Results are shown as mean ± standard deviation (n = 4) and were analyzed via one-way ANOVA followed by Tukey’s post hoc test. *, *** *p* < 0.05 and 0.001 in comparison to control groups; # *p* < 0.05 in comparison to TNFα-treated group. Permeability value of controls = 0.65 ± 0.09 × 10^−3^ cm/min.

The BBB model was shown to suffer alterations due to contact with peripheral inflammatory factors from IBD patients and healthy donors, even at low levels, TNFα being an important role player among these. Alterations caused, however, were minimal and led to no changes on permeability. As effects of larger magnitude knowingly disrupting the BBB would be better suited for assessment of the actions of pioglitazone, BLECs were treated with TNFα at a higher concentration following pre-treatment with pioglitazone in a manner mimicking intense peripheral inflammation. Under such circumstances, TNFα increased permeability to NaFlu, an effect attenuated by pioglitazone (Figure 3A). Cytoskeleton staining revealed that endothelial cells shrank or became elongated, indicating their organization as a barrier that might have been compromised (Supplementary Figure S2A–D), but such effects were not reliant on cell death (Supplementary Figure S2E). TNFα treatment also decreased expression of ZO-1 and claudin-3 while increasing expression of claudin-5, effects not influenced by pioglitazone, meaning pioglitazone is capable of preserving BBB integrity albeit not relying on modifying junctional protein expression. No changes were observed on VE-cadherin expression in any treatment condition (Figure 3B–F).

### 2.3. TNFα-Induced Disarrangement of Claudin-5 Is Attenuated by Pioglitazone

**Figure 4:**
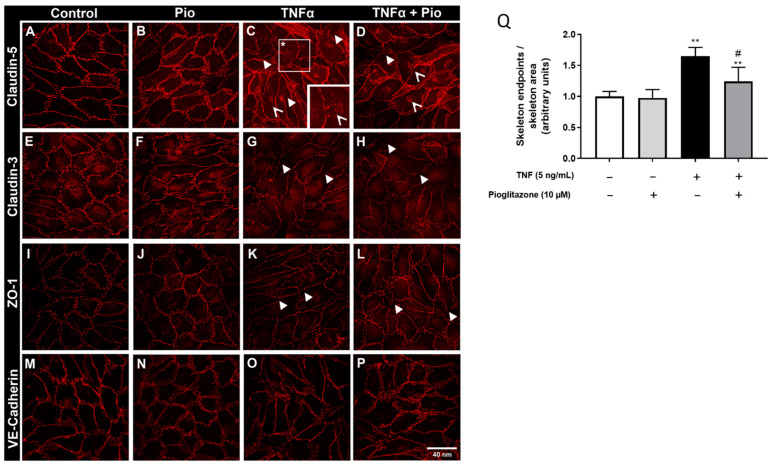
**Effects of pioglitazone on TNFα-induced disarrangement of junctional proteins.** BLECs were treated with 5 ng/mL of TNFα for 24 h following pre-treatment with 10 µM pioglitazone for 24 h. Filters from transwell inserts were collected, antibody-stained and mounted on glass slides. Visual inspection of organization of junctional proteins was assessed by immunofluorescence under a confocal microscope (100×) (**A**–**P**). Immunofluorescence images of claudin-5 were processed on CellProfiller^®^ software, and skeleton endpoint ratios (total “filled” skeleton area by the actual skeleton area) were assessed (**Q**). Scale bar = 40 nm. Thick white arrowheads represent segments of claudin-5 discontinuation; thin white arrowheads represent points of claudin-5 resorption; the image at the corner in C represents a higher magnitude (500×) amplifying the area denoted by a white box containing an asterisk. Results are shown as mean ± standard deviation (n = 4) and were analyzed via one-way ANOVA followed by Tukey’s post hoc test. ** *p* < 0.01 in comparison to control groups; # *p* < 0.05 in comparison to TNFα-treated group.

As pioglitazone treatment exerted no influence on junctional protein expression while still improving BBB permeability after TNF treatment, we hypothesized whether pioglitazone could modify the arrangement of these junctional proteins. Confocal immunofluorescence analysis showed, as expected, that treatment with TNFα compromised the organization of claudin-5, claudin-3 and ZO-1, there being increased disruption of protein fluorescence signal alongside cell–cell contact areas and even evidence of resorption of degraded claudin-5. Pioglitazone treatment, however, noticeably preserved claudin-5 arrangement, decreasing its discontinuity alongside cell–cell contact areas and seemingly reducing its resorption. There were no noticeable alterations in VE-cadherin arrangement (Figure 4A–P). As to further quantify the extent of pioglitazone effects, further analysis of skeletonized claudin-5 images revealed an increased amount of claudin-5 “breaking” sites alongside tight junction areas, effects attenuated by pioglitazone treatment (Figure 4Q), evidencing that pioglitazone does not necessarily control BBB permeability by modulating junctional protein expression, but rather by preserving their arrangement, claudin-5 being a major target for such actions.

### 2.4. Monocyte-Recruiting Alterations on the Inflamed BBB Are Halted by Pioglitazone

**Figure 5:**
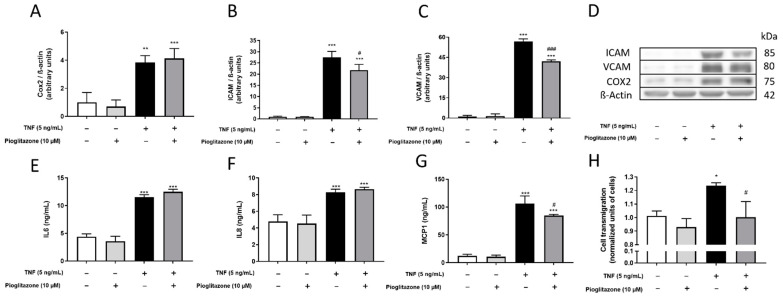
**Effects of pioglitazone on TNFα-induced increases in inflammation-associated factors and monocyte transmigration.** BLECs were treated with 5 ng/mL of TNFα for 24 h following pre-treatment with 10 µM pioglitazone for 24 h. Expression of inflammatory proteins COX-2, VCAM-1 and ICAM-1 were assessed by Western blotting (**A**–**D**). Co-culture supernatants were collected, and secretion of IL-6, IL-8 and MCP1 by endothelial cells was assessed by ELISA (**E**–**G**). Stained monocytes were co-cultured alongside BBB co-cultures, and their transmigration was assessed via colorimetric assays (**H**). Results are shown as mean ± standard deviation (n = 3) and were analyzed via one-way ANOVA followed by Tukey’s post hoc test. *, **, *** *p* < 0.05, 0.01 and 0.001 in comparison to control groups; #, ### *p* < 0.05 and 0.001 in comparison to TNFα-treated group.

As pioglitazone preserved BBB permeability and organization of junctional proteins, we next investigated whether it could also act on other inflammatory parameters that could directly be linked to a high-permeable, non-organized barrier structure, namely recruitment and transmigration of inflammatory peripheral leukocytes. After TNFα treatment, increased expression of COX-2 and secretion of IL-6 confirmed the inflamed status of BLECs, effects not attenuated by pioglitazone. TNFα treatment also increased expression of adhesion molecules VCAM-1 and ICAM-1 and increased secretion of IL-8 and MCP1, which are neutrophil/lymphocyte- and monocyte-attracting chemokines, respectively [19]; all such effects were attenuated by pioglitazone treatment except for IL-8 secretion (Figure 5A–G). These results indicate that pioglitazone is not capable of attenuating the effects of a peripheral inflammatory factor such as TNFα on the BBB as a whole, but given reductions in MCP1 secretion and ICAM/VCAM expressions, rather regulates a specific mechanism of BBB and subsequent CNS damage, which is recruitment and passage of peripheral monocytes through the endothelial barrier. In order to test this, monocytes were placed in contact with BLECs after treatments had been carried, and their passage was monitored. While TNFα treatment increased monocyte transmigration through the inflamed BBB, pre-treatment with pioglitazone reduced their passage to almost basal levels (Figure 5H).

### 2.5. TNFα-Induced Phosphorylation of ERK and NF-kB Is Attenuated by Pioglitazone

**Figure 6:**
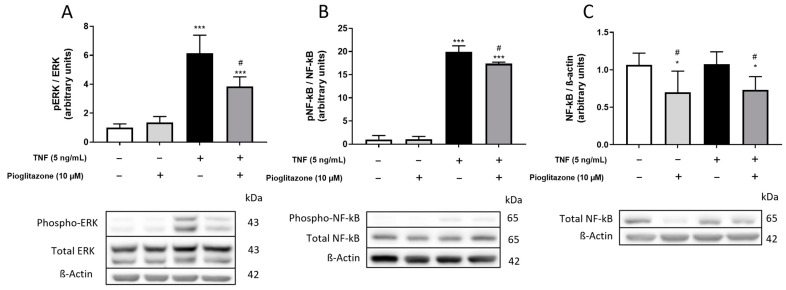
**Effects of pioglitazone on TNFα-induced phosphorylation of ERK and NF-kB.** BLECs were treated with 5 ng/mL of TNFα for 30 min following pre-treatment with 10 µM pioglitazone for 24 h; phosphorylation of ERK and NF-kB was assessed by Western blotting (**A**,**B**). Endothelial cells were also treated with 5 ng/mL of TNFα for 24 h rather than 30 min following pre-treatment with 10 µM pioglitazone for 24 h; expression of total NF-kB was assessed by Western blotting (**C**). Results are shown as mean ± standard deviation (n = 4) and were analyzed via one-way ANOVA followed by Tukey’s post hoc test. Representative bands are aligned with treatment order as denoted below graphs. *, *** *p* < 0.05 and 0.01 in comparison to control groups; # *p* < 0.05 in comparison to TNFα-treated group.

Phosphorylation of extracellular kinase (ERK) and nuclear factor kappa B (NF-kB) are known events downstream of TNFα receptor activation in the BBB leading to inflammatory effects, including secretion of inflammatory cytokines, expression of adhesion molecules and leukocyte recruitment [20,21,22]. We then investigated whether such signaling pathways could be related to pioglitazone actions, while a short-period TNFα treatment of BLECs increased phosphorylation of both ERK and NF-kB, pre-treatment with pioglitazone attenuated this effect (Figure 6A,B). Complementing these results, treatment of BLECs with pioglitazone for the full 48 h according to the overall experimental design rather than for only 24 h, as employed for protein phosphorylation, also led to degradation of total NF-kB, decreasing the amount of protein eligible for phosphorylation and further preventing downstream inflammatory signaling (Figure 6C).

### 2.6. Pioglitazone Reduces ICAM-1 Expression on Plasma-Treated BBB

**Figure 7:**
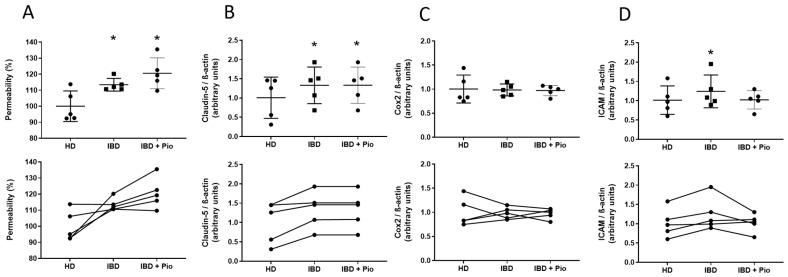
**Effects of pioglitazone on BBB alterations induced by plasma from IBD patients.** BLECs were treated with plasma from healthy donors and IBD patients at a 10% concentration for 24 h following pre-treatment with 10 µM pioglitazone for 24 h. Permeability to NaFlu through the endothelial barrier (**A**) and expression of junctional proteins claudin-5 (**B**), inflammatory protein COX-2 (**C**) and adhesion molecule ICAM-1 (**D**) by Western blotting were assessed. Results from an experiment utilizing 5 different donor pairs (n = 5) are shown as mean ± standard deviation at the upper brackets and as paired associations in the lower bracket. Data were analyzed via repeated-measures ANOVA followed by Dunn’s post hoc test. * *p* < 0.05 in comparison to healthy donor group. HD, healthy donor plasma; IBD, patient plasma; IBD + Pio, patient plasma plus pioglitazone added during the treatment. Permeability value of controls = 0.58 ± 0.050 × 10^−3^ cm/min.

After the effects of pioglitazone on TNFα-treated BLECs had been thoroughly investigated, we revisited our initial patient screening data aiming to verify whether it would be possible to investigate the effects of pioglitazone on BBB parameters when under stimulation by a sum of peripheral inflammatory factors found in IBD patient plasma, not limited to TNFα, and corroborating the mechanisms evidenced thus far under a proof of concept. For such, we selected patients whose plasma led to pronounced permeability alterations in the BBB model, ensuring that other inflammatory parameters would also be altered in an appreciative magnitude, regardless of correlations with TNFα levels. To further ensure that these alterations would be corroborated, the selection of plasma was also narrowed into patients in the worst clinical conditions suffering from disease flares and under therapeutic failure. A total of five IBD patients met these conditions and were selected for testing. Treatment of BLECs with plasma from such patients in comparison to treatment with plasma from matched healthy donors increased BBB permeability and expression of claudin-5 and ICAM-1, with COX-2 suffering no alterations. While treatment with pioglitazone prior to treatment with IBD patient plasma led to no alterations in permeability and claudin-5 expression, ICAM-1 expression was decreased (Figure 7A–D). These data suggest that pioglitazone can attenuate early inflammatory damage on the BBB elicited by peripheral inflammatory factors, not limited to TNFα, during progression of IBD, likely influencing ICAM-1 mediated effects.

## 3. Discussion

Investigation of the effects of anti-inflammatory drugs on the BBB while being used for treatment of other peripheral inflammatory conditions, given the rise of CNS disorders throughout the course of chronic inflammatory diseases, could prove valuable in better guiding pharmacotherapy of chronic inflammatory diseases by adding another layer of pharmacological targets to be addressed, namely the BBB, in the larger frame of peripherally induced CNS disorders [23]. In this context, chronic inflammation in IBDs has been increasingly linked to neuroinflammation, contributing to the onset of CNS diseases, but further studies on the effects of peripheral inflammation arising from IBDs on the BBB, even more so in human models, are lacking [12,13]. Previous data from our and other groups have also evidenced that pioglitazone is capable of halting inflammation in mice models of experimental colitis [1,15], and while there is evidence that PPARγ is also an important role player in BBB physiology [24], there are no studies on the anti-inflammatory actions of such ligands on the BBB as well.

By treating BLECs in our fully human in vitro BBB model with plasma obtained from IBD patients and matched healthy donors, we confirmed that the BBB model to be sensible to peripheral inflammatory factors at low levels. TNFα in plasma from both types of donors correlated positively with claudin-5, claudin-3 and ICAM-1 expressions. Still, BBB permeability was found to not be linked to either TNFα or any other parameter assessed; such a finding is reasonable, however, because cytokine levels found in donor plasma were way lower than what would be required for severe damage to occur to the BBB that would compromise its integrity. It is therefore expected that plasma from the majority of donors would not greatly modify BBB permeability, as if that were the case, patients would be severely crippled due to BBB damage [25]. Plasma from IBD patients showed overall lower levels of TNFα in comparison to their healthy counterparts, likely due to patients being treated with a myriad of anti-inflammatory drugs. This is a limitation of the study, as it is very difficult to enroll untreated IBD patients; due to symptoms, they are usually treated even before diagnosis, and responses to therapy can vary from failure to remission [26]. Alongside the long-spanning, intermittent aspect of such diseases, it can be challenging to correlate clinical disease severity with other blood parameters. Still, while TNFα is a major cytokine responsible for peripheral inflammatory damage during the course of IBDs, several other cytokines and inflammatory mediators influence overall inflammation in these conditions alongside TNFα [27].

As permeability is such an important factor correlating with BBB integrity, we decided to increase TNFα concentrations under controlled conditions to ensure that effects on permeability and other parameters would be of appreciable magnitude allowing for investigation of the anti-inflammatory actions and mechanisms of pioglitazone [28]. Under such experimental conditions, pioglitazone was effective in preventing TNFα-elicited increases in permeability by preserving claudin-5 organization while not affecting expression of any of the junctional proteins investigated. Claudin-5 is perhaps the most important component of tight junctions at the BBB. Studies show that BBB, in claudin-3 and occludin-deficient mice, despite functionality being compromised to an extent, retains its integrity, while mice lacking claudin-5 display a completely dysfunctional BBB [29,30,31]. In addition, corroborating our findings where claudin-5 expression is increased both by TNFα and by donor plasma, it is reported that its expression can increase due to acute inflammatory stimuli paralleled by decreased expression of other junctional proteins, especially in cases of systemic inflammation, likely in an attempt of the BBB to preserve its integrity when facing an inflammatory challenge, as claudin-5 is probably the most relevant tight junction protein in preserving BBB homeostasis [32,33]. Still, not only the expression of junctional proteins is important to assess BBB integrity, their organization and arrangement throughout cell–cell contact areas is equally important and might not necessarily correlate with quantitative expression; for instance, studies evidence in mice models that inflammation generated after stroke can dysregulate claudin-1 expression, compromising incorporation of claudin-5 into tight junction complexes. Likewise, our results showing that pioglitazone preserved claudin-5 arrangement while not influencing its expression corroborates this notion [34,35].

Following the investigation of other inflammatory effects on the BBB caused by TNFα, we found increased expression of Cox-2, VCAM-1 and ICAM-1 and increased secretion of IL-6, IL-8 and MCP1, with pioglitazone being capable of reversing the effects seen for VCAM-1, ICAM-1 and MCP1 only. At first, some of these are seemingly contradicting effects, as pioglitazone was reported in other studies to reduce levels of both interleukins and Cox-2 alongside VCAM-1 and ICAM-1, but these were reported under different conditions in different body compartments [36,37,38,39]; these results, while being reported on endothelial cells, are still far from the BBB model here used. As there are no studies on the actions of pioglitazone on inflammatory effects on the BBB for the sake of comparison, it is reasonable to assume, under the conditions here employed, that pioglitazone does not modulate inflammation in all of its aspects in the BBB. Instead, as such drugs managed to decrease expression of adhesion molecules and secretion of monocyte-attracting chemokines, it is likely that pioglitazone in the BBB is rather involved specifically with control of recruitment, adhesion and transmigration of peripheral leukocytes. Considering that a decrease in MCP1 levels (monocyte-attracting chemokine) rather than IL-8 levels (neutrophil/lymphocyte-attracting chemokine) was detected, it can be suggested that pioglitazone affects the recruitment of monocytes, specifically, rather than of other peripheral leukocytes [40].

Either way, it was confirmed that all of the aforementioned effects culminate in TNFα-induced increased transmigration of monocytes through the BBB model, while pioglitazone prevented their passage. This reveals a mechanism on how pioglitazone controls aspects of inflammation in the BBB, building on a series of small effects that synergize with one another and leading to the final effect of preventing passage of inflammatory cells into the brain, which is knowingly associated with neuroinflammation [41,42]. Adhesion molecules at the BBB are not only responsible for leukocyte adhesion, as they can also disorganize actin filaments binding to tight junction proteins, increasing endothelial barrier permeability [43]. In the same vein, MCP1 not only attracts peripheral monocytes, but also contributes in “opening” the BBB, favoring further transmigration, as it compromises expression and organization of junction proteins such as claudin-5 and ZO-1, also increasing endothelial barrier permeability [44]. Thus, alongside its effects on claudin-5, rather than controlling secretion of inflammatory cytokines, pioglitazone actions focus on regulating BBB disruption and transmigration of monocytes, preventing this manner of additional brain damage.

Expanding on these findings, pioglitazone prevented activation of ERK and NF-kB signaling pathways under the conditions tested. These are knowingly associated with neuroinflammation, compromising BBB integrity and favoring leukocyte transmigration, such as seen in meningitis and in autoimmune encephalomyelitis [20,21,22]; in endothelial progenitor cells in vitro, NF-kB activation is known to induce expression of both VCAM-1 and ICAM-1 [45]. Pioglitazone also induced cleaving of the NF-kB protein after a longer treatment period; it was previously reported that pioglitazone activates PPARγ, inducing its binding to the p65 subunit of NF-kB and leading to its degradation via ubiquitination [46]. Overall reduction of NF-kB expression following treatment with anti-inflammatory compounds in a glioblastoma cell line has also been reported, further reinforcing the notion that control of NF-kB expression is a relevant anti-inflammatory mechanism in TNFα-associated neuroinflammation not only in the BBB, but in the SNC as a whole [47,48]. Pioglitazone can thus not only prevent the phosphorylation of ERK and NF-kB, halting immediate downstream signaling, but it can also induce degradation of NF-kB, in turn preventing further inflammatory stimuli to activate such signaling pathways and causing BBB disruption.

Finally, by revisiting our patient data and treating endothelial cells with plasma from patients at severe clinical conditions alongside pioglitazone, we found that pioglitazone was able to attenuate increased ICAM-1 expression but not permeability or claudin-5 expression. While results for claudin-5 corroborate the previously assessed role of pioglitazone on TNFα-activated BBB, the lack of effect on permeability seemingly contradicts such results, but even if plasma from the patients being more severely affected by IBD was used, permeability increases were not higher than 10%, an effect likely not pronounced enough for pioglitazone actions to be detected. No changes in COX-2 levels also corroborate the notion that plasma from patients cannot elicit an inflammatory response as strong as TNFα at high concentrations, but ICAM-1 increases still indicate that damage is occurring. ICAM-1 might even be a better parameter to assess BBB damage, as studies in mouse models report that ICAM-1 expression correlates with changes in BBB permeability and that it increases as early as a few hours after peripheral inflammation is elicited, even before there is a significant increase in circulating inflammatory cytokines, meaning that ICAM-1 is very sensible to minimal peripheral inflammatory circumstances. These effects are also accompanied by an increased number of macrophages in brain tissue [49,50,51]. As pioglitazone was able to attenuate the increase in ICAM-1 promoted by patient plasma, it can be suggested that this PPARγ ligand is capable of preventing not only severe BBB damage as assessed on the experiments utilizing TNFα, but also in preventing early BBB damage occurring during low-grade inflammation as assessed during the experiments utilizing plasma from IBD patients at severe disease stages. This notion has been supported by other animal studies that reported that pioglitazone is effective in combating early inflammation and halting disease onset in ischemia and Parkinson’s disease animal models [52,53].

Overall, our findings show that peripheral inflammation is capable of disrupting BBB integrity at different magnitudes in a fully human in vitro BBB model utilizing primary cells, with pioglitazone attenuating associated effects.

## 4. Materials and Methods

### 4.1. Human BBB Model Derived from Hematopoietic Stem Cells

The BBB model used in this study consists in cultivating human primary endothelial cells derived from hematopoietic stem cells expressing CD34+ with human brain pericytes for 6 days. Endothelial cells acquire major BBB properties such as low paracellular permeability, expression of receptors and transporters, among others, and can be used to predict brain distribution of drugs or assess BBB physiology and neuroinflammation [54,55,56]. Under these conditions, endothelial cells are renamed brain-like endothelial cells (BLECs).

#### 4.1.1. Isolation and Culture of Human Endothelial Cells

The human BBB model has previously been described in detail [57]. Endothelial cells (ECs) were derived from CD34+ hematopoietic stem cells isolated from human umbilical cord blood according to a previously published method [58]. Written and informed consent from the donor’s parents was obtained for the collection of umbilical cord blood, in compliance with French legislation. Once isolated from umbilical cord blood, CD34+ cells were differentiated in vitro into endothelial cells (ECs) using endothelial cell growth medium (EGM; Lonza, Walkersville, MD, USA) containing 50 ng/mL vascular endothelial growth factor (PeproTech, Rocky Hill, NJ, USA) and 20% heat-inactivated fetal calf serum (FCS; Sigma Aldrich, St. Louis, MO, USA). After 15 to 20 days, ECs, now visible in culture dish, were then trypsinized and expanded in 0.2% (*w*/*v*) gelatin-coated 100 mm Petri dishes (Corning Inc., Corning, NY, USA) in endothelial cell medium (ECM; Sciencell, Carlsbad, CA, USA) supplemented with 5% (*v*/*v*) FCS, 50 µg/mL gentamycin (Biochrom AG, Berlin, Germany), and 1 ng/mL basic fibroblast growth factor (Sigma Aldrich).

#### 4.1.2. Co-Culture and Brain-like Endothelial Cells

Our syngeneic BBB in vitro model is based on the coculture of human CD34+-derived ECs with the human brain pericytes, instead of the initially described bovine ones [57]. Human brain pericytes were isolated, cultured and immortalized from brain tissue of a patient who suddenly died from a heart attack, as described previously [59]. The study protocol for human tissue was approved by the ethics committee of the Medical Faculty, University of Yamaguchi Graduate School and was conducted in accordance with the Declaration of Helsinki, as amended in Somerset West in 1996. Written informed consent was obtained from the family of the participant before entering the study.

Two days after thawing and growing in petri dishes, 5 × 10^4^ cells/cm^2^ human brain pericytes were seeded in 12 well-plates coated with rat tail collagen (type I)-coated (BD Biosciences, Franklin Lakes, NJ, USA), and cultured in basal endothelial cell medium ECM (Sciencell) supplemented with 5% FCS (ECM-5), 1% endothelial cell growth supplement (Sciencell) and 0.5% gentamicin (Biochrom AG), and kept for 3 h at 37 °C. To set up the syngeneic contact co-culture model, 0.4 µm transwell inserts (Corning Inc.) were coated with matrigel (BD Biosciences) diluted 1/48 (*v*/*v*). CD34+-derived ECs were seeded on the coated inserts at a concentration of 8 × 10^4^ cells/mL and immediately cocultured with the previously seeded brain pericytes. Cocultures remained in a humidified 5% CO_2_ atmosphere in ECM-5 medium, for 6 days. The medium was renewed every 2 days. After this period, ECs acquire a BBB phenotype as previously demonstrated [56,60,61] and become BLECs.

For transmigration experiments, CD34+-derived ECs were seeded on 3 µm transwell inserts (Corning Inc., Corning, NY, USA). To avoid unintended cell migration through pores and formation of double BBB layers on transwell inserts, we used a modified protocol we previously developed [62].

### 4.2. Collection of Blood from IBD Patients and Healthy Donors

Blood from a total of 30 IBD patients was collected in the period from January to April 2019 at the Santa Casa of Misericórdia of São Paulo, Brazil. All patients had been diagnosed with either UC or CD and had been followed by clinicians for a minimum of 2 years up to 15 years. Patients were informed of research details and asked on willingness to donate blood when coming to the hospital for follow-ups or for receiving pharmacotherapy, and those that agreed signed a form attesting their participation was of free-will and that they had been clarified on research purposes. Blood was then collected by venous puncture in sterile tubes containing heparin. Right after collection, blood was centrifuged at 3000 rpm for 15 min for separation of blood fractions. Plasma was then stored in sterile collection tubes at −80 °C until further cytokine analysis or use for in vitro cell treatment. Blood from 30 healthy donors matched by age and sex was also collected.

### 4.3. Cell Treatments

After acquiring BBB phenotype, BLECs had their culture medium changed and were treated with pioglitazone (Sigma Aldrich) at a concentration of 10 µM for 24 h. After this period, cells were treated with either TNFα (Sigma Aldrich) at a 5 ng/mL concentration or with plasma from donors at a 10% concentration for further 24 h alongside pioglitazone, amounting to a total of 48 h pioglitazone treatment. After this period, BLECs in filters were washed with phosphate buffer saline (PBS) and used for further permeability assessment, monocyte transmigration assays or fixed for immunofluorescence. Protein and RNA from BLECs were also obtained for further Western blotting and qPCR analysis. Medium supernatant from the apical side of cocultures was collected for assessment of released cytokines. In the case of TNFα treatment, cells were also treated in a different setting for 30 min after pioglitazone treatment for assessment of protein phosphorylation. All treatments were performed at the apical side of BLEC cultures, mimicking both pioglitazone and inflammatory mediators reaching the BBB peripherally through the bloodstream. Untreated cells received dimethyl sulfoxide (DMSO) at a concentration of 0.04%, which was the vehicle utilized for pioglitazone dilution.

The final experimental concentrations of human plasma, TNFα and pioglitazone were chosen based on permeability assays (details on next section) testing different concentrations of each. The chosen concentrations were a middle-ground between mild and toxic effects, resulting in effects of quantifiable magnitude. Pioglitazone concentrations were also chosen based on previous work [15]. Standardization tests are shown in Supplementary Materials (Supplementary Figure S3A–C).

The effect of treatments on cell viability was assessed via resazurin assay, as described elsewhere [63].

A graphical representation of the experimental design can be found in Supplementary Materials (Supplementary Figure S4).

### 4.4. Sodium Fluorescein (NaFlu) Permeability Coefficient (Pe_NaFlu_)

Endothelial permeability coefficient (Pe) of sodium fluorescein (NaFlu) was calculated as previously described [64].

HEPES-buffered Ringer’s solution was added to empty wells in a 12-well plate. Filter inserts containing BLECs were subsequently transferred to the 12-well plate and filled with Ringer-Hepes buffer (RH) containing the fluorescent integrity marker sodium fluorescein (NaFlu; 10 µM; Life Technologies, Carlsbad, CA, USA), which poorly crosses the BBB. After 1 h, filter inserts were withdrawn from the receiver compartment. Aliquots from the donor solution were taken at the beginning and at the end of the experiments, and the fluorescence was quantified using a microplate reader (Synergy H1^®^, BioTek, Colmar, France) at 490/525 nm.

The cleared volume was obtained by dividing the amount of NaFlu in the receiver compartment at the end by its concentration in the donor compartment at the start of the experiment; the result being further divided by the duration of the experiment (60 min) generates the permeability surface area product (PS, μL per minute). In this calculation, both the permeability of filters without cells (PSf = insert filter + coating) and of filters with cells (PSt = filter + coating + ECs) were taken into account, according to the formula:

1/PSe = 1/Pst − 1/Psf

PSe is the permeability surface area product of the ECs monolayer (in μL per minute) which is divided by the surface area of the filter (S, which is 1.13 cm^2^ for inserts which fit 12-well plates).

Finally, to generate the EC permeability coefficient (Pe, in cm/min), the PSe value was divided by the surface area of the porous membrane of the insert (1.12 cm^2^).

### 4.5. Western Blotting

After treatments, cells were collected in RIPA buffer (Millipore, Burlington, MA, USA) containing protease and phosphatase inhibitor cocktails (Sigma Aldrich). Cell lysates were centrifuged at 10,000 rpm for 10 min at 4 °C, and protein concentrations were determined by BCA assay (Biorad, Hercules, CA, USA), according to manufacturer instructions.

Aliquots of protein homogenates (20 µg of protein) were mixed with Laemmli reagent (Biorad), boiled at 95 °C for 5 min and subjected to protein separation by sodium dodecyl-sulfate polyacrylamide gel electrophoresis (SDS-PAGE) in 12% acrylamide gels (Biorad) at 200 V for 45 min. Proteins were then transferred to nitrocellulose membranes (GE Healthcare, Chicago, IL, USA) for 100 V for 1 h. After transferring, membranes were blocked with tris-buffer saline buffer containing 1% Tween 20 (TBS-T) and 5% skimmed milk for 1 h and finally incubated with primary antibodies at 4 °C overnight. Then, membranes were washed again with TBS-T and incubated with HRP-conjugated secondary antibodies (Dako/Agilent, Santa Clara, CA, USA) at a 1/5000 dilution at room temperature for 1 h. After development of membranes with enhanced-chemiluminescence reagents (GE Healthcare), images were acquired with a WB Imaging System Azure c600 (Azure Biosystems, Dublin, CA, USA), and later quantified utilizing ImageJ^®^ [65]. Intensities of protein bands are expressed as normalized optometric density units relative to ß-actin protein expression.

Primary antibody details are shown in Supplementary Materials (Supplementary Table S1). Representative images of uncropped, full blots for all targets assessed are shown in Supplementary Figure S5.

### 4.6. Conventional and Confocal Immunofluorescence Analysis

Arrangement and organization of junctional proteins were assessed by immunofluorescence microscopy. Transwell filters containing BLEC monolayers were fixed with ice-cold methanol after treatments for 1 min. Filter membranes were then rinsed with PBS for 5 min (3×), cut from the transwell inserts and blocked with Seablocking^®^ buffer (Thermo-Fisher, Waltham, MA, USA) for 30 min. Filters were rinsed again with PBS and incubated with primary antibodies diluted in PBS + 2% normal goat serum for 1 h at room temperature. After further washes, filters were then incubated with secondary polyclonal antibodies conjugated to fluorochromes (Life Technologies) at a 1/500 dilution for 30 min at room temperature in the dark. After a final set of washes, filters were placed atop of glass slides and mounted under coverslips with ProLong Gold Antifade Mountant^®^ containing DAPI (Thermo-Fisher).

Slides were analyzed in a Axio Imager A2^®^ fluorescence microscope (Leica Microsystems, Wetzlar, Germany) and in a Zeiss LSM-780-NLO^®^ confocal fluorescence microscope (Carl Zeiss, Oberkochen, Germany). Representative images from five different fields were obtained at 100× magnification for each slide, for each different marker.

Continuity of claudin-5 expression alongside cell–cell contact areas was assessed utilizing Cell Profiller 4.2.1^®^ software (Stirling et al., 2021). Pipelines for analysis were created adapting similar methods as described elsewhere [66].

Primary antibody details for IF are shown in Supplementary Materials (Supplementary Table S2).

### 4.7. ELISA

Cell supernatants from the apical side of co-cultures were collected for further assessment of cytokines via ELISA. Plasma from donors was also assessed for cytokine levels. Analyses were carried out utilizing BD Opteia^®^ (BD Biosciences) and R&D Systems ELISA kits (R&D Systems, London, UK) according to manufacturer instructions. Interleukin-6 (IL-6), interleukin-8 (IL-8) and monocyte chemoattractant protein 1 (MCP1) were assessed in culture supernatants, while TNFα, MCP1, interferon γ (IFNγ), metalloproteinase 9 (MMP-9) and tumor growth factor ß (TGF-ß) were assessed in donor plasma.

### 4.8. Monocyte Culture and Transmigration Assay

For transmigration assays, U937 monocytes obtained from the Rio de Janeiro Cell Bank (BCRJ) were cultured in RPMI (Sciencell) containing 10% FCS (Sigma Aldrich). Cells were grown in suspension and were centrifuged, washed with PBS and had their medium changed every 2–3 days.

Prior to transmigration assays, U937 monocytes were washed with serum-free RPMI (Sciencell), and a volume of 20 × 10^6^ cells was centrifuged at 1500 rpm for 5 min. Cell pellet was resuspended with 1.5 µM of Deep Red (Sigma Aldrich) solution prepared into serum-free RPMI (Sciencell) and kept under incubation at 37 °C for 45 min for staining. Prior to transmigration assay, stained monocytes were washed with PBS, centrifuged and resuspended into phenol red-free ECM (Sciencell) containing 0.1% BSA (Sciencell) at 2 × 10^6^ cells/mL.

Co-cultures prepared in 3 µm filters rather than in 0.4 µm filters had medium removed from the apical side and the filter immediately transferred to another well containing phenol red-free ECM containing 5% FCS (Sciencell). Stained U937 cells in a final amount of 1 × 10^6^ cells per 500 µL were placed atop of the BLEC monolayers. This system was allowed to rest inside an incubator for a period of 6 h so that transmigration could occur due to gradient difference.

After the migration period was over, medium aliquots were collected from the basolateral side of co-cultures and absorbance was measured utilizing a Synergy H1^®^ microplate reader (BioTek) at 660 nm. A standard curve was prepared utilizing serial dilutions of Deep-Red stained monocytes (highest point being 2 × 10^6^ cells/mL, lowest point being 0.015 × 10^6^ cells/mL), and number of transmigrated cells was then assessed according to the absorbance determined by the standard curve.

### 4.9. Bioinformatics

For visualization of data generated after treatment of BLECs with plasma from both healthy donors and IBD patients in a comparative manner, bubble heat-maps were generated utilizing Morpheus software (Morpheus, https://software.broadinstitute.org/morpheus, accessed on 1 March 2022). Results are shown as log2 fold-changes of data generated from treatment with patient plasma in comparison to data generated from treatment with healthy donor plasma.

Exploratory analysis of parameters assessed after treatment of BLECs with plasma from IBD patients alongside plasma from healthy donors was carried out by principal component analysis (PCA). Loading vectors and PCA bi-plots were shown to indicate correlation of parameters with principal components and data clustering, respectively. Likely correlations between assessed parameters, as indicated by orientation of PCA loading vectors, were further confirmed by Spearman’s correlation analysis, and results were compiled in a correlation matrix [67]. Analyses were performed utilizing R packages factoextra (version 1.0.7), FactoMineR (version 2.4) and corrplot (version 0.92).

### 4.10. Statistical Analysis

All data were analyzed with GraphPad Prism 7^®^ software (Graphpad Software^®^, San Diego, CA, USA). Normality of large patient datasets (n > 30) was assessed with the Kolmogorov–Smirnov test. Following comparisons between matched datasets were performed with Wilcoxon signed-rank test, and associations between two variables were assessed with Spearman’s correlation test. All other analyses of smaller datasets (n < 6) of either matched or non-matched data were performed utilizing one-way analysis of variance tests (ANOVA) followed by Tukey’s post hoc tests or repeated-measures ANOVA followed by Dunn’s post hoc tests. Results of analyses of parametric data are shown as mean ± standard deviation, while results of analyses of non-parametric data are shown as median alongside 0.25 and 0.75 interquartiles. Assessed parameters were considered significant when *p* < 0.05; values close to this threshold are reported where applicable.

## 5. Conclusions

Data obtained in the present work show that pioglitazone preserves BBB integrity via claudin-5 relocalization and prevents monocyte passage elicited by peripheral inflammation through it, as assessed in a fully human in vitro BBB model. Pioglitazone effects also seem to rely on attenuating TNFα-induced phosphorylation of ERK and NF-kB. These findings evidence pioglitazone as a potential drug capable of halting BBB inflammation elicited by peripheral inflammation, further preventing the onset of neurodegenerative disorders should it be used alongside other well-established drugs for treatment of chronic peripheral inflammatory diseases, such as IBD. These results also provide a solid foundation for further studies on PPARγ ligands to be carried out within this context.

## Figures and Tables

**Table 1:** **Descriptive Data of Healthy Donors and IBD Patients.** CD, Crohn’s disease; UC, ulcerative colitis. Failure or remission as indicated for CD patients refers to infliximab therapy. Medication listed as “others” refers to other drugs of minor relevance for IBD, such as antihypertensives and anti-diabetics.

Parameters	Healthy Donors	CD/Remission	CD/Fail	UC
Average age (min. − max.)	39 (21 − 60)	36.2 (19 − 53)	38 (19 − 63)	48
Standard deviation	12.2	12.0	13.9	7.2
Females	17	6	8	3
Males	13	4	7	2
Total	30	10	15	5
Medication				
Infliximab	0	10 (100%)	15 (100%)	5 (100%)
Corticosteroids	0	2 (20%)	7 (47%)	1 (20%)
Mesalazine	0	7 (70%)	9 (60%)	2 (40%)
Azathioprine	0	5 (50%)	7 (47%)	1 (20%)
Anti-diarrheic	0	0	2 (13%)	0
Anti-depressants	3 (10%)	1 (10%)	1 (7%)	0
Antibiotics	0	0	2 (13%)	1 (20%)
Others	6 (20%)	4 (40%)	11 (73%)	0

**Table 2:** **Levels of cytokines in healthy donor and patient plasma.** Cytokine levels in plasma of both healthy donors and IBD patients were measured by ELISA. Results obtained from analysis of plasma of n = 30 IBD patients and 30 matched healthy donors via Wilcoxon signed-rank test; *p* value considered significant when <0.05.

Cytokine	Healthy Donors	IBD Patients	*p* Value
TNFα (pg/mL)	6.12 (4.61; 8.25)	4.37 (3.83; 5.46)	0.0009
TGFß (ng/mL)	7.35 (4.12; 9.92)	5.71 (2.15; 12.62)	0.64
IFNg (pg/mL)	17.35 (14.01; 24.18)	18.03 (16.51; 21.53)	0.70
MCP-1 (pg/mL)	90.57 (70.28; 117.4)	83.51 (68.44; 107.8)	0.31
MMP-9 (ng/mL)	39.67 (32.95; 59.15)	26.88 (17.56; 46.83)	0.0093

## Data Availability

The data that supports the findings of this study are available on request from the corresponding author.

## Supplementary Materials

The following supporting information can be downloaded at: https://www.mdpi.com/article/10.3390/ijms232112781/s1.
