# Supplementary material for: Pioglitazone Attenuates the Effects of Peripheral Inflammation in a Human In Vitro Blood–Brain Barrier Model
Source: Int J Mol Sci. 2022 Oct 24;23(21):12781. doi: 10.3390/ijms232112781 (PMC9656730; doi:10.3390/ijms232112781)
Supplement: Supplementary file 1 [file ijms-23-12781-s001.zip › Supplementary Tables and Figure S3.pdf]

Supplementary Table S1 – WB antibody references

Target	Dilution	Reference	Supplier
Claudin-3	1/1000	Ab214487	Abcam
Claudin-5	1/1000	Ab15106	Abcam
Cox-2	1/500	AF4198	R&D Systems
ERK	1/1000	9102	Cell Signaling
pERK	1/1000	9106	Cell Signaling
ICAM-1	1/1000	Abs53013	Abcam
NF-kB	1/1000	Ab32536	Abcam
pNF-kB	1/500	Mab72261	R&D Systems
VCAM-1	1/1000	Abs98954	Abcam
VE-Cadherin	1/1000	Ab33168	Abcam
ZO-1	1/1000	Ab216880	Abcam
ß-actin	1/10000	A5541	Sigma Aldrich

Supplementary Table S2 – IF antibody references

Target	Dilution	Reference	Supplier
Claudin-3	1/100	341700	Invitrogen
Claudin-5	1/100	341600	Invitrogen
VE-Cadherin	1/400	Ab33168	Abcam
ZO-1	1/200	617300	Invitrogen
Vimentin	1/100	14989782	eBiosciences

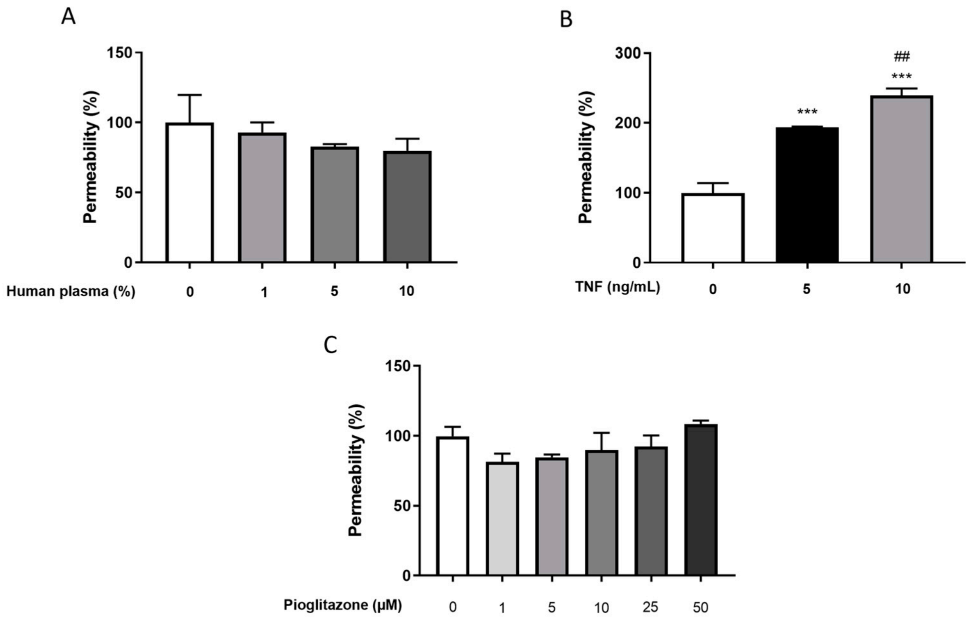

Supplementary Figure S3. Standardization of human plasma, TNFα and pioglitazone concentrations. Endothelial cells were treated for 24h with either 1, 5 or 10% plasma from a random healthy donor (A); 5 or 10 ng/mL of TNFα (B); or either 1, 5, 10, 25 or 50 μM of pioglitazone (C) and assessed for permeability to NaFlu. Results are shown as mean ± standard deviation (n = 3) and analyzed via Student's t test or One-Way ANOVA followed by Tukey's post-hoc test. *** $p < 0.001$ in comparison to control group. ## $p < 0.01$ in comparison to group treated with 5 ng/mL TNFα. Permeability value of controls in graphs A, B and C, respectively: 0.66 ± 0.13 x 10^{-3} cm/min, 0.64 ± 0.09 x 10^{-3} cm/min and 0.72 ± 0.04 x 10^{-3} cm/min
