# Supplementary figures and images for: Pioglitazone Attenuates the Effects of Peripheral Inflammation in a Human In Vitro Blood–Brain Barrier Model
Source: Int J Mol Sci. 2022 Oct 24;23(21):12781. doi: 10.3390/ijms232112781 (PMC9656730; doi:10.3390/ijms232112781)

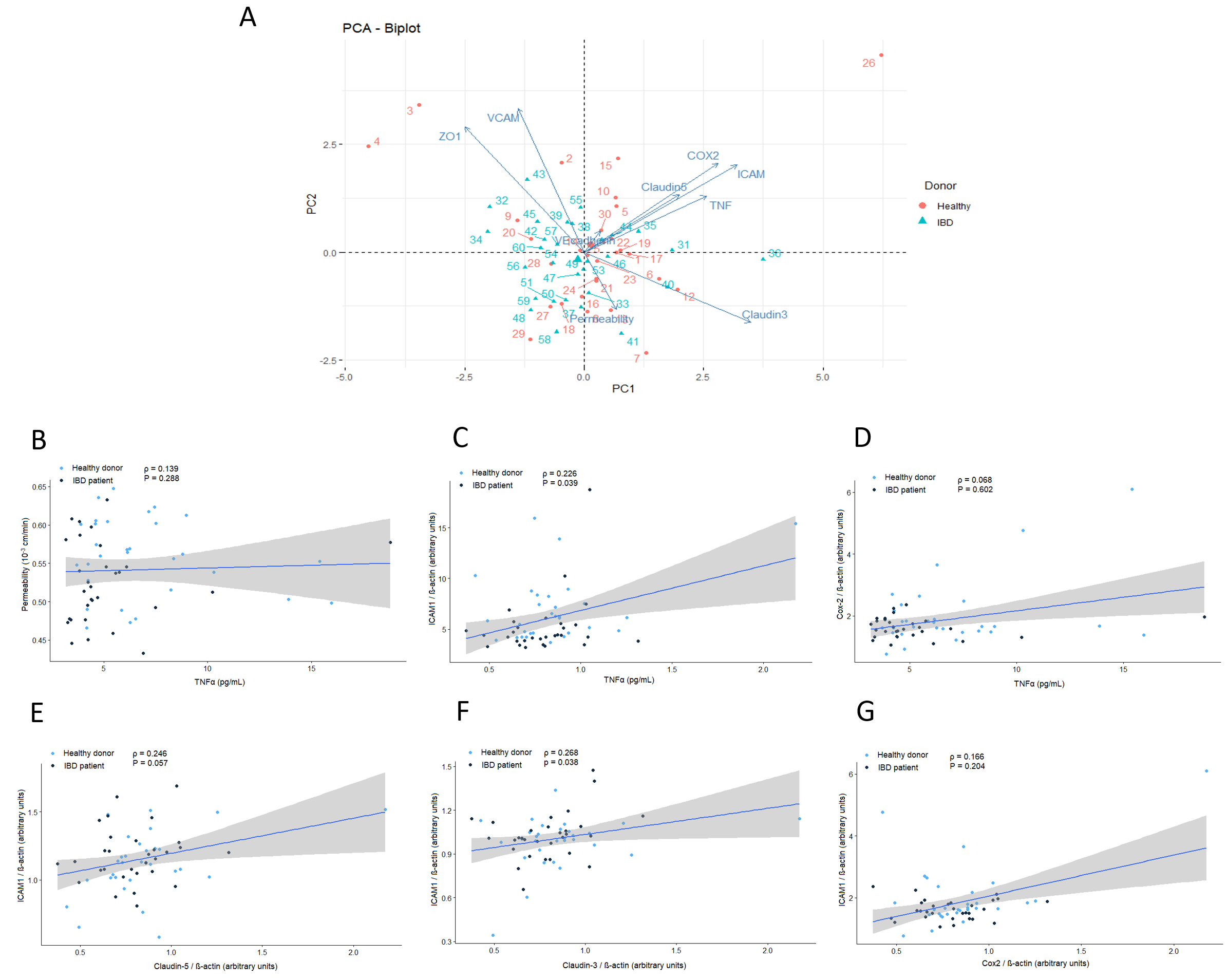

Supplement: Supplementary file 1 [file ijms-23-12781-s001.zip › Supplementary Figure S1.tif]

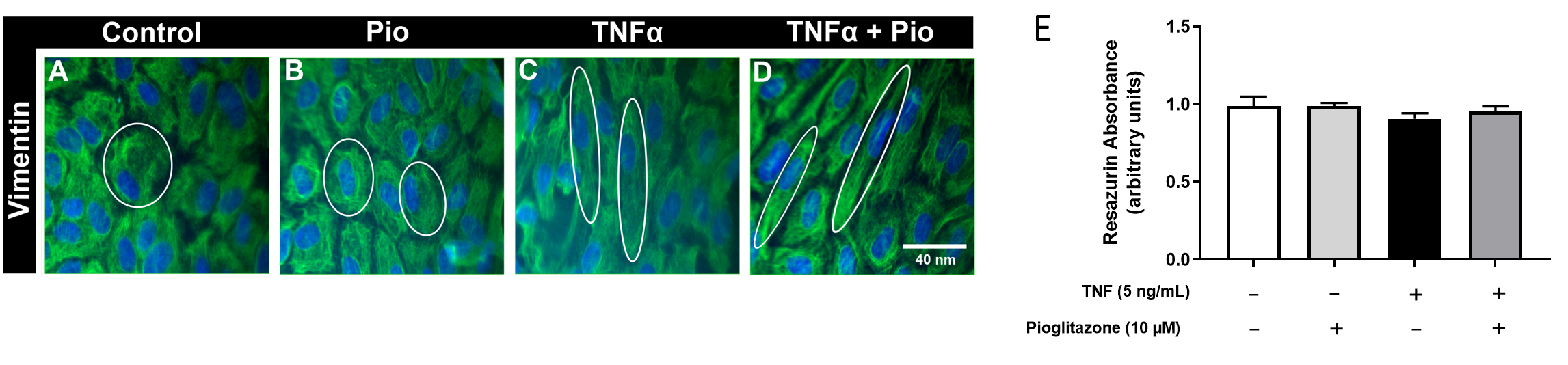

Supplement: Supplementary file 1 [file ijms-23-12781-s001.zip › Supplementary Figure S2.tif]

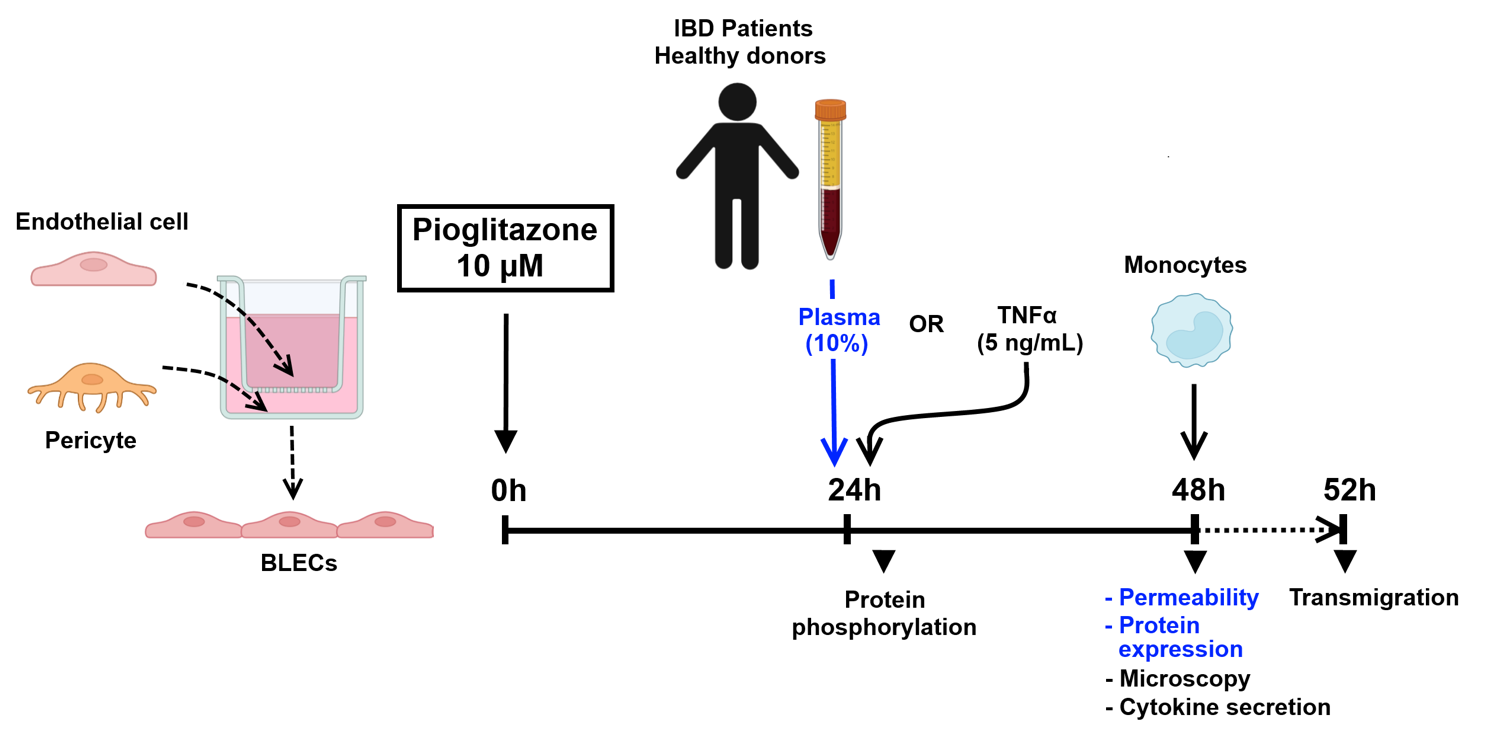

Supplement: Supplementary file 1 [file ijms-23-12781-s001.zip › Supplementary Figure S4.tif]

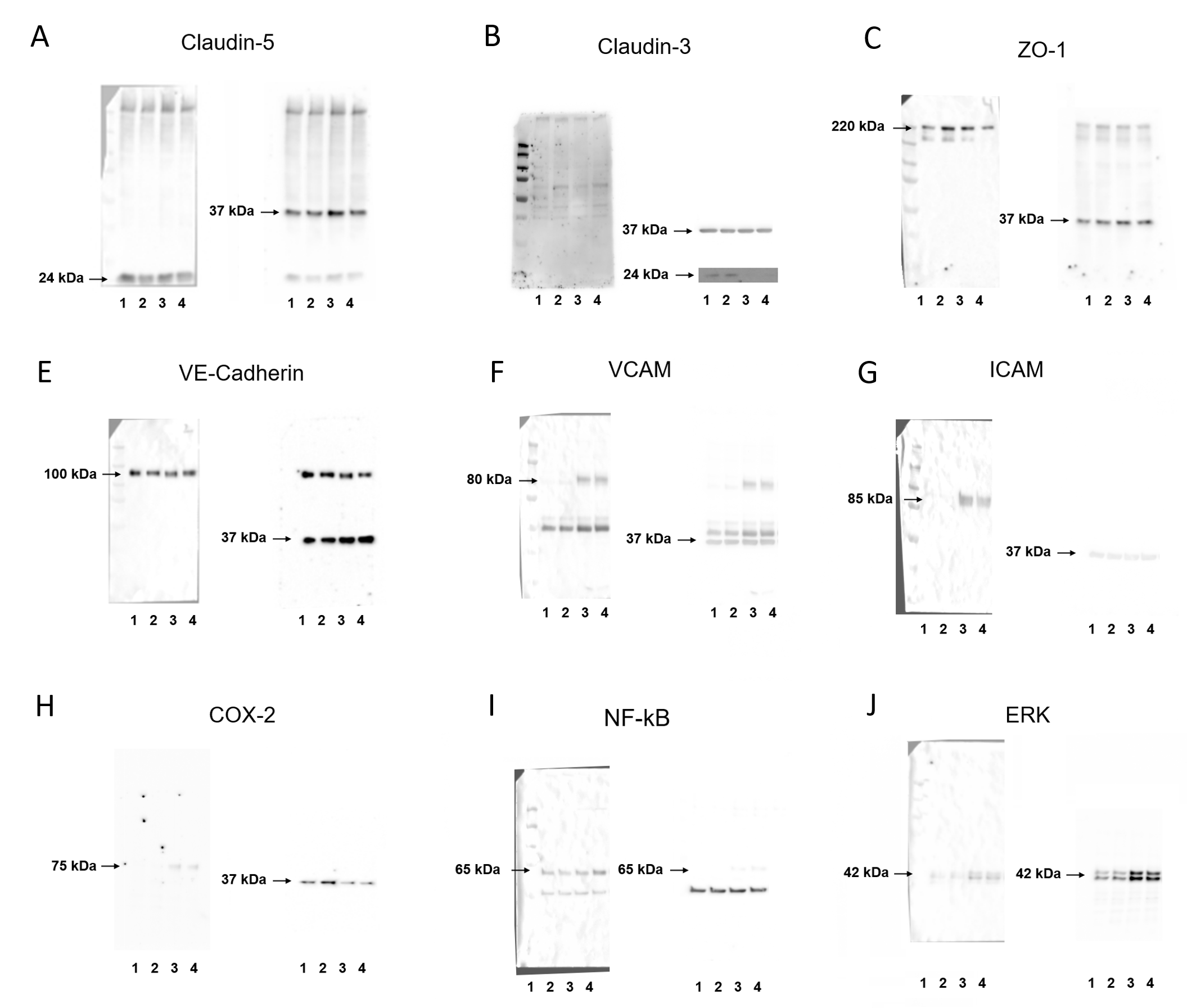

Supplement: Supplementary file 1 [file ijms-23-12781-s001.zip › Supplementary Figure S5.tif]
